# The TOSCA Registry for Tuberous Sclerosis—Lessons Learnt for Future Registry Development in Rare and Complex Diseases

**DOI:** 10.3389/fneur.2019.01182

**Published:** 2019-11-13

**Authors:** Ruben Marques, Elena Belousova, Mirjana P. Benedik, Tom Carter, Vincent Cottin, Paolo Curatolo, Maria Dahlin, Lisa D'Amato, Guillaume Beaure d'Augères, Petrus J. de Vries, José C. Ferreira, Martha Feucht, Carla Fladrowski, Christoph Hertzberg, Anna C. Jansen, Sergiusz Jozwiak, John C. Kingswood, John A. Lawson, Alfons Macaya, Finbar O'Callaghan, Jiong Qin, Valentin Sander, Matthias Sauter, Seema Shah, Yukitoshi Takahashi, Renaud Touraine, Sotiris Youroukos, Bernard Zonnenberg, Rima Nabbout

**Affiliations:** ^1^Novartis Farma S.p.A., Origgio, Italy; ^2^Institute of Biomedicine (IBIOMED), University of Leon, León, Spain; ^3^Research and Clinical Institute of Pediatrics, Pirogov Russian National Research Medical University, Moscow, Russia; ^4^SPS Pediatrična Klinika, Ljubljana, Slovenia; ^5^Tuberous Sclerosis Association, Nottingham, United Kingdom; ^6^Hôpital Louis Pradel, Claude Bernard University Lyon 1, Lyon, France; ^7^Tor Vergata University Hospital, Rome, Italy; ^8^Karolinska University Hospital, Stockholm, Sweden; ^9^Association Sclérose Tubéreuse de Bourneville, Gradignan, France; ^10^Division of Child and Adolescent Psychiatry, University of Cape Town, Cape Town, South Africa; ^11^Centro Hospitalar Lisboa Ocidental, Lisbon, Portugal; ^12^Universitätsklinik für Kinder-und Jugendheilkunde, Vienna, Austria; ^13^Associazione Sclerosi Tuberosa ONLUS, Milan, Italy; ^14^European Tuberous Sclerosis Complex Association, In den Birken, Datteln, Germany; ^15^Vivantes-Klinikum Neukölln, Berlin, Germany; ^16^Pediatric Neurology Unit, Department of Pediatrics, UZ Brussel VUB, Brussels, Belgium; ^17^Department of Child Neurology, Warsaw Medical University, Warsaw, Poland; ^18^Department of Neurology and Epileptology, The Children's Memorial Health Institute, Warsaw, Poland; ^19^Cardiology Clinical Academic Group, Molecular and Clinical Sciences Research Centre, St Georges University of London, London, United Kingdom; ^20^The Tuberous Sclerosis Multidisciplinary Management Clinic, Sydney Children's Hospital, Randwick, NSW, Australia; ^21^Hospital Universitari Vall d'Hebron, Barcelona, Spain; ^22^Institute of Child Health, University College London, London, United Kingdom; ^23^Department of Pediatrics, Peking University People's Hospital, Beijing, China; ^24^Tallinn Children Hospital, Tallinn, Estonia; ^25^Klinikverbund Kempten-Oberallgäu gGmbH, Kempten, Germany; ^26^Novartis Healthcare Pvt. Ltd., Hyderabad, India; ^27^National Epilepsy Center, Shizuoka Institute of Epilepsy and Neurological Disorders, NHO, Shizuoka, Japan; ^28^Department of Genetics, CHU-Hôpital Nord, Saint Etienne, France; ^29^St. Sophia Children's Hospital, Athens, Greece; ^30^University Medical Center, Utrecht, Netherlands; ^31^Department of Pediatric Neurology, Necker Enfants Malades Hospital, Imagine Institute, Inserm U1163, Paris Descartes University, Paris, France

**Keywords:** lessons, issues, strengths, weaknesses, TOSCA, registry, TSC

## Abstract

**Introduction:** The TuberOus SClerosis registry to increase disease Awareness (TOSCA) is an international disease registry designed to provide insights into the clinical characteristics of patients with Tuberous Sclerosis Complex (TSC). The aims of this study were to identify issues that arose during the design, execution, and publication phases of TOSCA, and to reflect on lessons learnt that may guide future registries in rare and complex diseases.

**Methods:** A questionnaire was designed to identify the strengths, weaknesses, and issues that arose at any stage of development and implementation of the TOSCA registry. The questionnaire contained 225 questions distributed in 7 sections (identification of issues during registry planning, during the operation of the registry, during data analysis, during the publication of the results, other issues, assessment of lessons learnt, and additional comments), and was sent by e-mail to 511 people involved in the registry, including 28 members of the Scientific Advisory Board (SAB), 162 principal investigators (PIs), and 321 employees of the sponsor belonging to the medical department or that were clinical research associate (CRA). Questionnaires received within the 2 months from the initial mailing were included in the analysis.

**Results:** A total of 53 (10.4%) questionnaires were received (64.3% for SAB members, 12.3% for PIs and 4.7% for employees of the sponsor), and the overall completeness rate for closed questions was 87.6%. The most common issues identified were the limited duration of the registry (38%) and issues related to handling of missing data (32%). In addition, 25% of the respondents commented that biases might have compromised the validity of the results. More than 80% of the respondents reported that the registry improved the knowledge on the natural history and manifestations of TSC, increased disease awareness and helped to identify relevant information for clinical research in TSC.

**Conclusions:** This analysis shows the importance of registries as a powerful tool to increase disease awareness, to produce real-world evidence, and to generate questions for future research. However, there is a need to implement strategies to ensure patient retention and long-term sustainability of patient registries, to improve data quality, and to reduce biases.

## Introduction

Patient registries are organized systems that use observational study methods to collect uniform data on a patient population defined by a particular disease, exposure or condition (e.g., age, pregnancy, specific patient characteristics), and which is followed over time (1). Patient registries may also help to understand the natural history of the disease, to estimate the human and economic burden of the disease, to monitor clinical practice patterns, to identify patients' subgroups that might be included in future clinical trials and to generate new research questions (2).

Therefore, patient registries are a key instrument to develop clinical research, and to improve patient care and healthcare planning, particularly in the field of rare diseases. In spite of its usefulness, patient registries do have several limitations arising from biases, lack of standardization in data collection, accuracy, and comprehensiveness of the data, fragmentation of clinical data, and ethical concerns (2). Most registries are carried out in a small number of centers belonging to a single country or, at best, in a limited number of countries (3), which constitutes an important limitation for the generalizability of the results. The fact that many registries are initiated in the field of academia might also limit their use for pharmaceutical research. In addition to academic initiatives on registries, there are different initiatives worldwide for patients' group registries where the accuracy of the data can be questioned.

The TuberOus SClerosis registry to increase disease Awareness (TOSCA) is a multicenter, international disease registry that was designed to assess manifestations, interventions, and outcomes in patients with Tuberous Sclerosis Complex (TSC), a rare genetic disorder characterized by growth of hamartomas in several organs (4). This registry, designed as an observational clinical study, enrolled from 2012 to 2014 a total of 2,216 patients in 170 sites in 31 countries worldwide. Patients of any age diagnosed with TSC having a documented visit for TSC within the preceding 12 months or newly diagnosed patients (4) were enrolled after signing an inform consent form (ICF) approved by local ethic committee (EC)/institutional review board (IRB). Patients' data were collected at baseline visit and at 5 yearly follow-up visits and recorded by principal investigators (PIs) in an electronic clinical database. The registry clinical database lock occurred in 2017.

The TOSCA registry design consisted of a main “core” part and a number of sub-studies (referred to as “research projects” or “petal projects”) (4). The “core” section was designed to collect a general predefined set of patient background data including demographics, family history, prenatal history, and disease features (i.e., neurological, neuropsychiatric, renal, cardiovascular, pulmonary). Additional and more detailed data related to specific disease manifestations were collected in the sub-studies/research projects of the registry. Additionally, the TOSCA registry included a sub-study designed as post approval safety study (PASS), following the European Medicines Agency's (EMA) request (EMEA/H/C/002311/II/0004), to document the long-term safety and tolerability profile of Votubia® in the treatment of TSC patients residing in the European Union for the licensed indications and collect everolimus therapeutic drug monitoring (TDM) data within routine clinical practice as per SmPC. Clinical study protocol and final study results are available on ENCePP portal at http://www.encepp.eu/ (EU PAS Register Number EUPAS324) (5).

The TOSCA registry was funded, designed and managed by a pharmaceutical sponsor (Novartis) with the support of a Scientific Advisory Board (SAB), a Working Committee (WC), and Research Groups (4):

- The SAB consisted of up to 30 members, including TSC healthcare professionals, patient representatives and a maximum of three representatives of the sponsor (Novartis). The medical experts were selected based on the number of publications in TSC, research interests and working in reference sites for TSC in their country. Patient representatives were included as well to ensure that their perspective is considered in the project design and execution. The chair and co-chair were selected by vote of all members. The SAB was responsible for the scientific principles of the registry, the promotion of the use of the registry, the publication of data, and the approval of research projects. All the details of SAB constitution, rules and goals are reported in a SAB charter.
- The WC was a subgroup consisting of up to 14 members from the SAB and was responsible for the registry content and coordination of all the operational activities, for defining the statistical analysis plan and publication policy, and for developing and maintaining the database structure of the registry. All the details of WC constitution, rules, and goals were reported in a WC charter.
- Research groups were made up of physicians participating in the registry and their role consists on the submission of research project proposals to the WC, together with the subsequent management of that particular project.

Apart from being the largest registry in patients with TSC, the TOSCA registry has noteworthy features, including its worldwide scope (including European and non-European countries), its nature as a large-scale cooperation effort between healthcare professionals, patient representatives and pharmaceutical industry, the inclusion of a large number of patients, the design as a core minimal set of data and the more detailed data collection on specific aspects (research projects), the long-term follow-up (up to 5 years), and the inclusion of a PASS sub-study (4). For this reason, both in terms of contents and structure, the TOSCA registry offers an excellent opportunity to assess what lesson can be learnt from a registry, which issues should be addressed and what pitfalls can be avoided when setting up and managing an international registry in a rare disease.

## Objective

The aim of this analysis was 2-fold: firstly, to identify issues that arose during the design and operation of the TOSCA registry and during the interpretation and publication of the results; secondly, it aimed to identify areas for improvement and pitfalls that can be useful for the development of successful future registries in rare and complex diseases.

This paper is structured as follows. Section Methods describes the methodology and the instruments employed to extract the information. Section Results describes the issues encountered by each group of stakeholders in every domain of the registry; it also outlines the pitfalls and lessons learnt from the integration of the research projects and the everolimus sub-study PASS within the TOSCA registry. Finally, section Discussion contains a discussion of the results and provides recommendations for future registries in rare, multisystemic, and complex diseases.

## Methods

A questionnaire was designed to identify issues that might have arisen at any stage of the TOSCA registry project from its inception to the publication of the results, and to identify its strengths and weakness, and opportunities and threats that could be of interest for the development of future registries in rare diseases. It was developed by the TOSCA clinical trial head with contribution of TOSCA patient representatives steering committee members and Novartis quantitative safety and epidemiology department. The questionnaire was built following a guide aimed to support the design, implementation, analysis, interpretation, and quality evaluation of registries published by Gliklich et al. (2). The questions included were prepared based on the steps to conduct a registry described in this guideline and the specific TOSCA registry project characteristics.

The questionnaire contained 225 questions split into seven sections (Supplementary Material); the first five sections covered a range of aspects related to issues during the registry (planning, operation, data analysis, results publication, and other issues), and the last two were devoted to assess lessons learnt from the TOSCA registry and to gather additional comments (Table 1).

**Table 1:** Structure of the Questionnaire.

**1) Identification of issues during registry planning** • Perception on the definition of the purpose and the objectives of the registry • Perception on the definition of the inclusion/exclusion criteria • Definition of the variables included in the registry • Definition of the size, the duration, the setting and the geographical areas • Identification of stakeholders, team building and establishment of a governance • Data access & use of data • Publication plan • Development of the protocol and related documents • Development of the project plan • Development of risk management plans & risk management during the registry
**2) Identification of issues during the operation of the registry** • Issues related to patient recruitment or retention ° Barriers to patient recruitment/retention ° Evaluation of success of patient recruitment strategies ° Evaluation of success of patient retention strategies ° Evaluation of center/physician or patient selection bias • Issues related to data collection & quality assurance ° Issues related to data collection ° Identification of quality issues & timing for detection • Issues related to budget • Issues related to project management ° Ownership & accountability ° Coordination ° Estimation of the use of resources/duration/complexity
**3) Issues during data analysis** • Identification of sources of bias • Treatment of missing data • Appropriateness of time horizon & planned interim analysis • Appropriateness of pre-specified analyses • Interpretation of the results • Identification of issues related to data access • Identification of strengths & limitations of the registry
**4) Issues during the publication of the results**
**5) Other issues**
**6) Assessment of learnings** • General learning topics • Value of the registry organization ° Inclusion of patients in the SAB and in the WC ° Inclusion of clinicians in the SAB and in the WC ° Inclusion of members from the pharmaceutical industry in the SAB and in the WC • Pitfalls and learning opportunities emerged from the integration of research projects within the TOSCA registry • Pitfalls and learning opportunities emerged from the integration of a Votubia^®^ PASS within the TOSCA registry
**7) Additional comments**

*SAB, Scientific Advisory Board; WC, Working Committee; TOSCA, TuberOus SClerosis registry to increase disease Awareness; PASS, post approval safety study*.

On September 7th 2018 the questionnaire was sent by e-mail to the 511 people who had been involved in the TOSCA registry. Twenty-eight of them were part of the SAB, while 162 were principal investigators (PIs) and 321 were Novartis employees not included in the SAB. All the receptors of the questionnaire (henceforth “participants”) received the same document, but some questions precluded respondents to answer subsequent parts of the questionnaire (for instance, if participants responded that were not involved in budget planning, allocation and/or control, they were invited to skip the subsequent questions regarding these topics). To facilitate the analysis, most questions were close-ended (“yes”/“no” or using a Likert scale). Besides, all the questions contained “N/A” (not applicable) option and a free-text field where the participants were encouraged to justify their answers. The participants were given 2 months for replying and two reminders were sent. No remuneration was offered to respondents.

The analysis was carried out on the completed questionnaires received in the 2 months following the initial mailing (cut-off date: November 8th 2018). All data were analyzed using Microsoft Excel. Relative and absolute frequencies were analyzed for all the questions, and whenever possible, for the groups of questions belonging to the same section or subsection.

## Results

By the cut-off date (November 8th 2018), a total of 53 questionnaires were received (53/511; 10.4%). The response rates per type of participant who filled the questionnaire in (hereafter referred to as “respondents”) were 64.3% (18/28) for members of the SAB including Novartis representatives, 12.3% (20/162) for PIs not included in the SAB and 4.7% (15/321) for other Novartis employees not included in the SAB.

The overall rate of completion of the questionnaire (i.e., answered questions/total questions) was 88% for closed questions (of the amount of missing data per question was 12% on average, range 2–30%); the rates of missing data according to the type of respondent were 4% for members of the SAB, 4% for PIs and 7% for other Novartis employees.

### Identification of Issues

**Figure 1:**
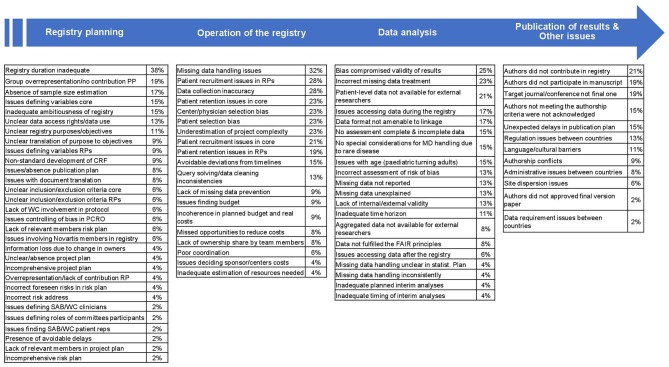
Typology and Weight of Issues derived from the Different Stages within the TOSCA Registry. CRF, case report form; FAIR, Findable, Accessible, Interoperable and Reusable (data); MD, missing data; PCRO, patient-caregiver reported outcomes; PP, project plan; RP, research project; SAB, Scientific Advisory Board; WC, Working Committee.

A summary of all the issues reported by the survey respondents in relation to TOSCA is shown in Figure 1. This figure represents the main stages of the TOSCA registry *(registry planning, operation, data analysis, publication, and other)* and the issues encountered by the respondents in each of these stages. Percentages in brackets are related to the proportion of respondents who reported each issue. Questions from the survey which were not rated as an issue by any of the respondents were not included in Figure 1. These non-issue questions mainly relate to the identification of clinicians to lead the research projects or to delays in the development of the registry due to patient identification. All respondents also agreed that no issues arose neither on the grade of involvement of WC members in the protocol and related documents, nor in the documentation of protocol amendments, nor whether the information about these amendments was provided in a timely manner to respondents. Finally, no issues were reported regarding registry oversight or the adverse event collection/reporting processes.

#### Registry Planning

The limited duration of the registry (up to 5 years) was considered the most common issue amongst the survey respondents (38%). There was a consensus amongst those answering the questionnaire on the appropriateness of having a long-term registry and some respondents stated that a longer follow-up would have been good in order to capture the impact of the disease in a more realistic way; however, constraints, such as budget limitations, were impactful leading to substantial amounts of missing data from follow-up 3. Respondents considered the registry too ambitious in terms of recruitment, duration or compliance and its long-term sustainability unrealistic. Conversely, timeline delays, risk, and project plan problems and issues when defining SAB-WC members were the lowest-rated complications associated with registry planning.

#### Operation of the Registry

Missing data were the main complication stated by respondents in relation to the operational domain of the registry (32%) (Figure 1). Variables with the most data missing were related to TSC-Associated Neuropsychiatric Disorders (TAND)—for reasons such as the lack of knowledge of these TSC manifestations by the physicians—or patient/caregiver reported outcomes, whereas those with fewer missing data were associated to physical signs and symptoms of the patients. A low proportion of respondents stated issues related to resources and costs and there were mainly related to budget limitations, especially toward the research projects.

#### Data Analysis

The effect of bias on the validity of the results was considered as the main issue related to data analysis by the respondents (25%), together with the incorrect treatment of missing data (stated by 23% of the respondents). More than half of the respondents (51%) agreed on the presence of some type of bias, either selection bias (e.g., unclear inclusion-exclusion criteria or registry population as a non-random selection from the target population), information bias (e.g., selective recall, inconsistent data collection, or wrong-inexact data recording) and/or measurement bias (e.g., faulty-inaccurate measurements or misclassification of outcomes). The involvement of statisticians throughout the whole project from its conception, budget extensions or further monitoring during data collection were considered as potential solutions to these issues by the respondents. Issues related to interim analyses and missing data handling were amongst the least reported by the respondents (4% of the respondents each issue) (Figure 1) in this section and mainly related to the desire of making these analyses longer and the missing data present in the final follow-ups (follow-up 4 and follow-up 5).

#### Publication of Results and Other Issues

Regarding publication of the results and other issues, the lack of contribution to the TOSCA registry and the lack of participation in manuscripts were the issues most rated by the respondents in the survey (21 and 19%, respectively), whereas questions related to data requirements between countries and final approval of publications were considered the less important complications related to the registry (2% of the respondents each issue). Overall, respondents felt that no authorship conflicts (e.g., issues related to the inclusion of all authors and/or the order in which some authors appeared in publications) happened during the publication process (<10% of respondents stated this type of issue).

### Assessment of Lessons Learnt From TOSCA Registry

Table 2 shows contributions of the TOSCA registry to the field of TSC and the rate of agreement of the respondents with these contributions. These contributions were classified into the ones finally accomplished by TOSCA registry and those not accomplished, either because it was not achieved even though it was intended or because it was not intended (Table 2). Overall, the rates of completeness were high in this section of the questionnaire, with an average rate of missing data of 5% per question (range 2–15%) mainly due to the fact that they did not remember the data or did not have access to it.

**Table 2:** Assessment of lessons learnt derived from the TOSCA registry (*N* = 53).

**TOSCA registry contributions**	**Yes**	**No, but it was intended**	**No, but it was not intended**	**Missing**	**N/A**
Improvement of knowledge on the natural history of TSC and its manifestations	47 (89%)	3 (6%)	0 (0%)	1 (2%)	2 (4%)
Increase disease awareness	46 (87%)	2 (4%)	1 (2%)	1 (2%)	3 (6%)
Identification of useful information for the development of clinical research in TSC	44 (83%)	2 (4%)	1 (2%)	3 (6%)	3 (6%)
Trigger research questions/developing hypothesis for new research in TSC	41 (77%)	2 (4%)	4 (8%)	3 (6%)	3 (6%)
Improvement of epidemiological knowledge of TSC	40 (75%)	2 (4%)	7 (13%)	1 (2%)	3 (6%)
Foster the communication between TSC experts and Novartis	40 (75%)	3 (6%)	4 (8%)	3 (6%)	2 (4%)
Improvement of knowledge on the clinical management of the disease in different countries	38 (72%)	3 (6%)	7 (13%)	1 (2%)	4 (8%)
Provision of data on quality of life	38 (72%)	3 (6%)	8 (15%)	2 (4%)	2 (4%)
Identification of useful information for the development of studies involving large/diverse geographic areas	38 (72%)	3 (6%)	6 (11%)	2 (4%)	3 (6%)
Foster the communication between TSC experts	38 (72%)	3 (6%)	7 (13%)	3 (6%)	2 (4%)
Provision of data on the effectiveness & efficiency of interventions in the real world	37 (70%)	7 (13%)	5 (9%)	2 (4%)	2 (4%)
Improvement of clinical practice	37 (70%)	4 (8%)	7 (13%)	2 (4%)	3 (6%)
Quantification of the use of resources and the burden of the disease	37 (70%)	7 (13%)	3 (6%)	2 (4%)	4 (8%)
Identification of centers/physicians treating patients with TSC	35 (66%)	5 (9%)	7 (13%)	4 (8%)	2 (4%)
Identification of useful information for the development of studies in pediatric patients	34 (64%)	3 (6%)	10 (19%)	2 (4%)	3 (6%)
Foster the communication between TSC experts and patients	34 (64%)	4 (8%)	7 (13%)	4 (8%)	3 (6%)
Assessment of the agreement between clinical practice and guidelines	33 (62%)	7 (13%)	9 (17%)	2 (4%)	2 (4%)
Provision of data on the safety of the interventions in patients with TSC in the real world	31 (58%)	7 (13%)	10 (19%)	3 (6%)	2 (4%)
Improvement of health care planning & resource allocation	31 (58%)	9 (17%)	8 (15%)	2 (4%)	3 (6%)
Development of new clinical practice guidelines	30 (57%)	8 (15%)	9 (17%)	3 (6%)	3 (6%)
Identification of patients with TSC that might benefit from certain interventions or might be included in future clinical trials	30 (57%)	8 (15%)	9 (17%)	3 (6%)	3 (6%)
Identification of useful information for the development of clinical research in other rare diseases	28 (53%)	6 (11%)	10 (19%)	4 (8%)	4 (8%)
Foster the communication between TSC patients and Novartis	24 (45%)	7 (13%)	12 (23%)	6 (11%)	3 (6%)
Facilitation of market access for Votubia®	23 (43%)	6 (11%)	10 (19%)	8 (15%)	4 (8%)

*TOSCA, TuberOus SClerosis registry to increase disease Awareness; TSC, Tuberous Sclerosis Complex*.

More than 80% of the survey respondents perceived that TOSCA improved the knowledge on the natural history and manifestations of TSC, increased the awareness of the disease and helped to identify information relevant to clinical research. Thus, overall there was a convergence that the TOSCA registry positively contributed to make progress into the knowledge of TSC, although one respondent considered this progress as small given the cost and time spent in the registry. The lowest consensus was reached on the items “the registry contributed to facilitate market access for Votubia®” and “the registry contributed to foster the communication between TSC patients and Novartis”, agreed by <50% of the respondents.

The items where TOSCA made no contribution to the fields of rare diseases registries or TSC were classified in those where the registry was not meant to contribute and those where the contribution was intended but not accomplished (Table 2). Fewer than 20% of respondents stated items where the contribution was intended but not accomplished, mainly in improving healthcare planning and resource allocation (17%) or developing new guidelines (15%). The items from which the contribution was not accomplished but also not intended were mainly related to foster the communication between TSC patients and Novartis (23%).

Most respondents considered the inclusion of different groups (TSC experts [reported by 84%], the pharmaceutical industry [reported by 75%] and patient representatives [reported by 59%]) in the SAB and the WC as either important or very important, despite some respondents were concerned that including patient representatives would create issues, such as ethical issues (reported by 6%) or confidentiality issues (reported by 6%). Overall, more than 75% of the respondents considered the inclusion of patient representatives to be good in facilitating communication—about the registry's purpose and value to patient advocacy groups—and to furthermore increase public awareness of the disease. Seventeen percent of the respondents also stated that they would have increased the number of patient representatives in the SAB/WC, especially if they had medical background.

There was a clear convergence regarding the importance of including TSC experts in the SAB and the WC, especially to provide interpretation of results, to propose the collection of variables and analyses of medical interest and to improve the quality of publications (more than 90% of respondents rated the inclusion of TSC experts as relevant or very relevant for these items). However, respondents considered the overall number of TSC experts to be too high in both in the WC and SAB. There was also agreement about the importance of including members of the pharmaceutical industry in the SAB and the WC, especially to provide technical, and/or financial support in the dissemination and publication of the results (rated as important or very important by more than 80% of respondents). However, the inclusion of different pharmaceutical companies as well as members with more specific skills (e.g., statistics, medical, operational, data management) was felt necessary by few respondents (9 and 2%, respectively).

#### Pitfalls and Lessons Learnt From the Integration of Research Projects Within the TOSCA Registry

More than half of the respondents (57%) considered appropriate to include research projects within the structure of the TOSCA registry. Further benefits derived from the projects were the extensive data collection and its multidisciplinary nature, which would have allowed a deep analysis of specific areas of TSC resulting in better knowledge of the disease, and furthermore the procurement of patient reported outcomes, such as burden of illness or quality of life.

On the other hand, respondents also stated that research projects were complex, burdensome and should have been considered at the registry planning stage (as they were included as study protocol amendments). The absence of publications and statistical plans together with the lack of budget (for aspects such edit checks on collected data or PI reimbursement for data entry) and patient retention were other pitfalls stated in the survey.

On average, 38% of respondents considered that separating the core from the research projects was a good idea; conversely, 17% of the respondents on average stated that this separation caused delays and agreed that both the core and the research projects should have been done simultaneously.

No consensus was reached regarding the efficiency in resource management for the research projects (28% of respondents considered the management efficient, whereas 23% thought it was not).

Regarding the contents of the core and the research projects, there were mixed opinions on whether some variables in the core registry should have been included in the research projects, and vice versa (21% said “yes” vs. 23% said “no,” 43% said “N/A,” 13% were missing). Regarding the amount of missing data, there was also an absence of consensus regarding whether the proportion of missing data was similar between the core and the research projects; missing data appeared to be reported similar between the core and the research projects by 18% of the respondents who provided a valid answer (e.g., yes, no or N/A), while considered different by 25%. The opinions reflected in the answers on whether the number of respondents in the research projects was sufficient to answer questions of clinical relevance were heterogeneous (19% said “yes” vs. 26% said “no”; 38% said “N/A”, 15% were missing). More consensus was obtained on the representativeness of the results, as 38% of the respondents providing a valid response stated that results from the research projects could be extrapolated to all the respondents in the core registry, and 43% stated that results from the research projects would be representative of real world.

Finally, more respondents agreed that research projects provided striking or relevant results (17% said “yes” vs. 13% said “no,” 51% “N/A”, 19% were missing) while there was uncertainty on whether new projects emerged from the research projects (13 vs. 11% said “yes” and “no,” respectively; 58% reported “N/A”, 17% were missing). Of those who stated that the research projects provided relevant findings, these were related to the impact on renal angiomyolipoma (rAML), the effects of subependymal giant cell astrocytoma (SEGA) in adults, the results obtained in TAND and aspects related to quality of life. Appropriateness in the dissemination of results was uncertain (19% said “yes”, 19% said “no”, 42% said “N/A”, 21% were missing).

#### Pitfalls and Lessons Learnt From the Integration Everolimus, Votubia® PASS (Post Authorization Safety Study) Within the TOSCA Registry

Some questions in the survey were related to the PASS study, which was embedded in the TOSCA registry to evaluate the long-term safety profile of everolimus (commercially known as Votubia®) an orphan drug directed to treat SEGA, rAML and seizures that did not respond to other treatments. Almost half of the respondents (43%) considered appropriate to integrate the PASS study within the TOSCA registry, mainly due to efficiency gains such as better surveillance, retention, recruitment, and long-term effects of adverse events. However, some pitfalls also emerged from this integration, as the extra workload imposed by PASS within TOSCA design, the characterization of PASS as a sub-study of TOSCA and the important differences between both studies (e.g., administrative, reporting, regulatory requirements).

Approximately 30% (range 26–34%) of respondents agreed on the convenience of separating the elaboration, data collection, and approval of both the PASS and TOSCA, and 32% of the respondents considered that there was a good management of time and resources in PASS.

Conversely to what happened with the research projects, more respondents considered that there were no variables in PASS that should have been collected in the core registry or vice versa (9 vs. 19%, on average). Twenty-one percent of the respondents considered data quality and completeness was worse in the TOSCA registry than in the PASS. There were discrepancies between respondents regarding the number of patients in PASS, with 13% of respondents thinking they were sufficient vs. 9% who considered the sample unrepresentative (60% said “N/A”, 17% were missing). A bigger proportion of the respondents considered the results in PASS representative of the whole TOSCA population (17%) and translatable into real world (25%) that those who did not (8 and 2%, respectively). Importantly, none of the respondents perceived that new projects emerged from the PASS study, although there was an important degree of uncertainty surrounding this item (19% said “no,” 62% reported “N/A,” 19% were missing).

Regarding the dissemination of results, respondents had mixed opinions (11% said “yes”, 8% said “no”, 62% reported “N/A”, 19% were missing). No consensus was reached regarding the potential benefit on the TOSCA registry derived by the interaction of health authorities during the PASS, again with important levels of uncertainty (8% said “yes”, 8% said “no”, 68% wrote “N/A”, 17% were missing).

## Discussion

The analyses performed here identified the main issues that arose during TOSCA registry from its inception to the publication of the results, and the take-home messages and lessons that could be relevant to the design and development of future registries in rare and complex diseases.

All the respondents agreed that one of the most positive aspects of the TOSCA registry was the involvement of a range of stakeholders (including TSC experts, members from industry, and patients). By involving people with different perspectives and profiles, the study analyzed variables that were of interest to physicians, to the pharmaceutical industry, and most importantly, to patients.

There is a growing emphasis on patient-focused registries (6) and, in this particular case, patients' representative in the SAB were considered a key element to facilitate communication of the results to advocacy groups, and to increase public awareness on the disease. Other successful examples of registries with an active participation of patients in its design, governance and/or operation are the ImproveCareNow network for inflammatory bowel disease in the United States (7), the ParkinsonNet Approach in the Netherlands (8), and the TREAT-NMD European network for neuromuscular disorders (9).

In the TOSCA registry, no issues were reported regarding registry oversight, adverse event collection/reporting processes (only related to the PASS sub-study), or project management, which means that these aspects worked particularly well. The use of standard operational procedures may have helped to prevent this type of issues and is highly advised for the development of future registries.

Another aspect that was rated positively was the high recruitment in the core project. The recruitment strategies varied among the enrolling countries and included phone contacts, proposal of participation in scheduled visits, exploitation of local patient databases, targeted mailing and newsletters to the investigators, virtual investigator meetings and the contacts with local patients' associations and family groups.

By contrast, patient retention was poor in TOSCA registry; after 3 years follow up, some sites stopped reporting data in a constant manner and a high number of patients discontinued (93.5%). Patient discontinuation is a common issue in all the registries. Therefore, strategies to reduce losses to follow-up are urgently needed, especially when taking into account that approximately a third of the respondents answered that they would have preferred the TOSCA registry to have a longer duration or even to be permanent.

The contrast between the low retention rates and the high expectations highlights the need for realistic goals when setting up a registry, but also the need for continuous motivation, adequate budget, and close oversight for registries that are expected to last longer than one or 2 years. Unfortunately, long-term sustainability is an important issue for most registries (1).

Issues related to missing data collection were among the most common difficulties during the operation of the registry and during data analysis, especially in the last follow-up visits. According to one of the respondents, carrying out a pilot study would have been useful to make sure questions were formulated in the most optimal way, and to reduce the amount of missing data. Other strategies related to missing data reduction or handling are to detail mechanisms to identify and collect missing data in the protocol, to distinguish between nice-to-have, and essential data (as in TOSCA study management document like the CRF manual and of monitoring plan) and to describe the handling of missing data in the statistical analysis plan (also part of TOSCA study management documents) (1).

Issues related to language translations were not observed in the TOSCA registry, which can be considered a success in a project involving 31 countries. Within the TOSCA registry, the impact of translation issues was minimized by several actions, such as the study oversight and site support provided in local languages including the discussion of the protocol and the electronic case report forms (eCRFs) requirements. In spite of this, one of the respondents mentioned that in any future multinational project, agreeing, and defining each term or concept with representatives from each country and language would be important to avoid any issue related to a mistranslation. These solutions might be useful for future multinational registries.

During data analysis, the most important issues were related to biases. Due to its observational nature, registries are prone to many biases. In this case, several respondents concluded that, due to selection bias toward patients with severe manifestations recruited in large hospitals and reference centers, the burden of the disease might have been overestimated. Another reason for selection bias was the overrepresentation of pediatric neurologists. Despite of the biases, the TOSCA registry provided relevant information about the presence of clinical manifestations on TSC patients such epilepsy that was useful from an epidemiological point of view. Besides, the eCRF included some specific questions for some specialties that could not be answered properly by all the participants; therefore, data collection for some specialties such as dermatology or ophthalmology was not completely reliable. Future studies should ensure that the sample is sufficiently homogeneous and representative of the population to be analyzed, that the investigators are a representative sample of the physicians treating that condition, and that all the variables can be properly assessed by the investigators involved in the study. Reducing bias therefore requires the participation of statisticians when planning the project, a careful site and PI selection across countries and also an increased and continuous support at site level to understand study requirements and eCRF questions. This issue was always specified in the different results and publications of the TOSCA registry, where it was emphasized that this is not an epidemiological study, but a very large cohort study.

Apart from potential biases and missing data issues, there were difficulties related to data access. In spite of the existence of a definition of the terms for data access, one TSC expert believed that the data access rights favored too much the sponsor and others thought that they were not clear enough. Therefore, more efforts are required to involve all the stakeholders in the definition of data access terms. In this respect, a discussion paper elaborated by the EMA Cross-Committee Task Force on Patient Registries goes even further, and acknowledges that “clarity is needed regarding data ownership, including patients' wishes regarding the use of their data” (1).

Issues during the publication of data from other registries have not been previously analyzed. Authorship conflicts were reported by 9% of the respondents. The most frequent issues were related to the poor involvement of some authors in the manuscripts or the lack of acknowledgment for all the contributors. This highlights the need for authorship criteria based on real contribution instead of pre-signed agreements.

Another conclusion resulting from analyzing the deviations between the planned and the expected journals for the publication is that setting unrealistic target journals might be an important cause for delays during the publication process. The difficulties related to publishing results from yearly follow-ups should also be taken into account when devising a publication plan.

According to most respondents, it was positive to carry out research projects besides the TOSCA registry because they allowed to carry out detailed analyses of specific manifestations in patients with TSC or provided additional information on the burden of the disease. However, due to insufficient funding and to the lack of specific statistical and publication plans, the validity and dissemination of the results from the research projects were scarce. In addition, most respondents considered that the research projects were not well-handled and that the implication from the investigators was not sufficient. This might be seen as a lost opportunity, but also as a need for better planning for studies emerging from registries, and highlights the need to include detailed budget planning within all project proposals. Interestingly, the EMA provided very clear guidance on this matter stating the importance of differentiating between registries (including their periodic analyses) and registry studies. In line, protocols are meant to be completely separate, meaning the addition of research projects as amendments are not in line with the Good Registry Practice and should be considered as almost separate studies with their own budget, management, monitoring, etc. (1).

Conversely, most respondents considered data quality and completeness were worse in the TOSCA registry than in the PASS. While it is true that the aims of a PASS study are completely different from those in the TOSCA registry, a better integration of the TOSCA registry and the PASS could have been exploited to increase the quality of the TOSCA registry.

The analysis of the lessons from TOSCA might also have some limitations. First, it is only based on one single registry experience in patients with a single disease. However, most of the issues are applicable to registries in other diseases. The second limitation is associated to the low number of TSC patients' representatives who were able to fill this questionnaire. This might be due to the low percentage of patient representatives in the SAB. Thirdly, a major limitation was the high percentage of the SAB in the respondents' group. Some reasons for the low response rates of the PIs and Novartis employees could be the perception on the burdensomeness of the questionnaire, the lack of economic compensation for the participants, a decreasing interest in the study or a lack of belief in the interest of such questionnaire. In future studies, a pilot of the questionnaire should be performed in a small sample of the population before being distributed further in order to test the validity and reliability of the questionnaire and to improve response rates.

Finally, the questionnaire was designed and sent 1 year after the completion of the registry, and this may have resulted in recall biases. In any case, we believe that by performing the analysis retrospectively, we could obtain a complete view on the difficulties arisen throughout the project.

In conclusion, this analysis has contributed to foresee and prevent issues in the design and development of future multinational registries in rare diseases. Careful planning, adequate monitoring and sufficient budget allocation are key elements for the success of registries. By contrast, there is a need to improve data quality, to reduce biases, to avoid access-related issues, and to ensure patient retention and long-term sustainability. Finally, this analysis also shows that registries are a powerful tool to increase disease awareness, and to produce a real-world view of clinical practice, but they have many limitations too. When designing and carrying out a registry, keeping a balance between ambition, pragmatism, and costs is a difficult task.

## Data Availability Statement

Novartis supports the publication of scientifically rigorous analysis that is relevant to patient care, regardless of a positive or negative outcome. Qualified external researchers can request access to anonymized patient-level data, respecting patient informed consent, contacting study sponsor authors. The protocol can be accessed through EnCePP portal http://www.encepp.eu/ (EU PAS Register Number EUPAS3247).

## Ethics Statement

The study protocol and all amendments were reviewed and approved (if applicable) by independent ethics committee/institutional review board for each centre. Written informed consent to participate in this study was provided by the participants' legal guardian/next of kin.

## Author Contributions

RM designing the study, data analysis, data interpretation, drafting, revising, final review, and approval of the manuscript. EB, MB, PC, MD, JF, MF, CH, SJ, JL, AM, RN, VS, MS, RT, BZ, JK, and AJ designing the study, patient accrual, clinical care, data interpretation, drafting, revising, final review, and approval of the manuscript. TC, GB, VC, PV, CF, FO'C, JQ, YT, and SY designing the study, data interpretation, drafting, revising, final review, and approval of the manuscript. LD'A designing the study, trial management, data collection, data analysis, data interpretation, drafting, revising, final review, and approval of the manuscript. SS designing the study, trial statistician, data analysis, data interpretation, drafting, revising, final review, and approval of the manuscript.

### Conflict of Interest

RM and SS are employees of Novartis, while LD'A was a Novartis employee at the time of manuscript concept approval. EB, TC, VC, PC, GB, PV, JF, MF, CF, CH, SJ, RN, FO'C, JQ, MS, RT, MD, JL, AM, SY, MB, BZ, JK, and AJ received honoraria and support for the travels from Novartis. VC received personal fees for consulting, lecture fees and travel from Actelion, Bayer, Biogen Idec, Boehringer Ingelheim, Gilead, GSK, MSD, Novartis, Pfizer, Roche, Sanofi; grants from Actelion, Boehringer Ingelheim, GSK, Pfizer, Roche; personal fees for developing educational material from Boehringer Ingelheim and Roche. PV has been on the study steering group of the EXIST-1, 2 and 3 studies sponsored by Novartis, and co-PI on two investigator-initiated studies part-funded by Novartis. RN received grant support, paid to her institution, from Eisai and lectures fees from Nutricia, Eisai, Advicenne, and GW Pharma. YT received personal fee from Novartis for lecture and for copyright of referential figures from the journals, and received grant from Japanese government for intractable epilepsy research. SJ was partly financed by the EC Seventh Framework Programme (FP7/2007–2013; EPISTOP, Grant Agreement No. 602391), the Polish Ministerial funds for science (years 2013–2018) for the implementation of international cofinanced project, and the grant EPIMARKER of the Polish National Center for Research and Development No. STRATEGMED3/306306/4/2016. JK, PC, CH, JL, and JQ received research grant from Novartis. VS reported no conflict of interest. This study was funded by Novartis Pharma AG. All authors approved the final version of the manuscript prior to submission.
