# Supplementary material for: The TOSCA Registry for Tuberous Sclerosis—Lessons Learnt for Future Registry Development in Rare and Complex Diseases
Source: Front Neurol. 2019 Nov 13;10:1182. doi: 10.3389/fneur.2019.01182 (PMC6863928; doi:10.3389/fneur.2019.01182)
Supplement: Supplementary file 1 [file Data_Sheet_1.PDF]

Supplementary Material

Questionnaire

Identification of issues during registry planning

Perception on the definition of the purpose and the objectives of the registry

1. Were you involved in the definition of the purpose and the objectives of the registry?

☐ **Yes** **Comments**
☐ **No**
☐ **N/A**

2. When looking at the registry retrospectively (*i.e.* now, at the end of the registry), do you think the purpose and objectives of the registry were clear enough? If not, why?

☐ **Yes** **Comments**
☐ **No**
☐ **N/A**

3. Do you think the purpose of the registry was clearly translated into specific objectives? If not, why?

☐ **Yes** **Comments**
☐ **No**
☐ **N/A**

4. Do you think the "ambitiousness" of the registry was adequate? If not, why?

☐ **Yes** **Comments**
☐ **No**
☐ **N/A**

5. If you had the possibility to carry out a similar registry in the future, would you somehow change the goals/objectives of the registry? If yes, why?

☐ **Yes** **Comments**
☐ **No**
☐ **N/A**

Perception on the definition of the inclusion/exclusion criteria

6. Were you involved in the definition of the inclusion/exclusion criteria of the registry?

☐ **Yes** **Comments**
☐ **No**
☐ **N/A**

7. When looking at the registry retrospectively (*i.e.* now, at the end of the registry), do you think the inclusion or exclusion criteria were clearly defined **in the core registry**? If not, why?

☐	**Yes**	**Comments**	
☐	**No**		
☐	**N/A**		

8. Do you think the inclusion or exclusion criteria were clearly defined **in the research projects**? If not, why?

☐	**Yes**	**Comments**	
☐	**No**		
☐	**N/A**		

9. If you had the possibility to carry out a similar registry in the future, would you change any of the inclusion/exclusion criteria in the core registry or in any research project? If yes, which one/s and why?

☐	**Yes**	**Comments**	
☐	**No**		
☐	**N/A**		

Definition of the variables included in the registry

10. Were you involved in the definition of the variables to be included **in the core registry**?

☐	**Yes**	**Comments**	
☐	**No**		
☐	**N/A**		

11. Were there any issues when defining the variables to be included in the core registry? If so, please detail.

☐	**Yes**	**Comments**	
☐	**No**		
☐	**N/A**		

12. Were you involved in the definition of the variables to be included **in any of the research projects**? If so, which research project?

☐	**Yes**	**Comments**	
☐	**No**		
☐	**N/A**		

13. Were there any issues when defining the variables to be included in the research projects? If so, please detail.

☐ **Yes** **Comments**
☐ **No**
☐ **N/A**

14. If you had the possibility to carry out a similar registry in the future, would you change any of the variables from the core data set or from the research projects? If, yes, which one/s and why?

☐ **Yes** **Comments**
☐ **No**
☐ **N/A**

Definition of the size, the duration, the setting and the geographical areas

15. Were you involved in the definition of the size, duration, setting or geographical areas of the registry?

☐ **Yes** **Comments**
☐ **No**
☐ **N/A**

16. Overall, do you think it was positive to include non-European countries in the registry? Why?

☐ **Yes** **Comments**
☐ **No**
☐ **N/A**

17. Was there an estimation of the registry sample size (overall) or of the expected sample size per country? If not, why?

☐ **Yes** **Comments**
☐ **No**
☐ **N/A**

18. Do you think the duration of the registry was adequate? If not why?

☐ **Yes** **Comments**
☐ **No**
☐ **N/A**

19. If you had the possibility to carry out a similar registry in the future, would you carry it out in a different setting (e.g. changing the target specialties or putting more emphasis on different specialties, reducing/widening the number of participants, etc.)? Why?

- ☐ **Yes** **Comments**
- ☐ **No**
- ☐ **N/A**

20. If you had the possibility to carry out a similar registry in the future, would you change (either reduce or widen) the geographical area? Why?

- ☐ **Yes** **Comments**
- ☐ **No**
- ☐ **N/A**

Identification of stakeholders, team building and establishment of a governance

21. Were you involved in the identification of any of the stakeholders of the project? If the answer is **No**, please skip questions 22-31.

- ☐ **Yes** **Comments**
- ☐ **No**
- ☐ **N/A**

22. Were there any difficulties when defining/finding the members from Novartis that would be involved in the registry? If so, please specify the issues. Please explain as well how Novartis stakeholders were identified.

- ☐ **Yes** **Comments**
- ☐ **No**
- ☐ **N/A**

23. Were there any changes in the registry project owners (either operational or scientific) during the development of the registry? If so, please explain how they were managed.

- ☐ **Yes** **Comments**
- ☐ **No**
- ☐ **N/A**

24. Did the changes in the registry project owners led to information loss? If so, please specify.

- ☐ **Yes** **Comments**
- ☐ **No**
- ☐ **N/A**

25. Were there any difficulties in the identification of the clinicians that would take part in the scientific advisory board (SAB) or in the working committee (WC)? If so, please specify.

☐	**Yes**	**Comments**	
☐	**No**		
☐	**N/A**		

26. Were there any difficulties in the identification of the clinicians that would lead the research projects? If so, please specify.

☐	**Yes**	**Comments**	
☐	**No**		
☐	**N/A**		

27. Were there issues when defining the roles of the participants in the committees? If so, please specify.

☐	**Yes**	**Comments**	
☐	**No**		
☐	**N/A**		

28. Were there any difficulties when finding patient representatives to be involved in the SAB and in the WC? If so, please specify.

☐	**Yes**	**Comments**	
☐	**No**		
☐	**N/A**		

29. Did the identification of any of the participants delay the development of the registry? If so, please specify.

☐	**Yes**	**Comments**	
☐	**No**		
☐	**N/A**		

30. Which aspects of the strategy for the identification of participants worked particularly well and might be useful for the design of future studies?

31. If you had the possibility to carry out a similar registry in the future, would you include additional stakeholders or would you introduce any other change to improve the identification of stakeholders?

☐	**Yes**	**Comments**
☐	**No**	
☐	**N/A**	

Data access & use of data

32. Were you involved in the definition of the terms and conditions for data access & use of data?

☐	**Yes**	**Comments**
☐	**No**	
☐	**N/A**	

33. Do you think data access rights and use of data were clearly defined during the registry design? Why?

☐	**Yes**	**Comments**
☐	**No**	
☐	**N/A**	

34. If you had the possibility to carry out a similar registry in the future, what would you change to improve the definition of the rights to data access or to the use of data and why?

Publication plan

35. Were you involved in the preparation of the publication plan? If the answer is **No**, please skip questions 36-38.

☐	**Yes**	**Comments**
☐	**No**	
☐	**N/A**	

36. Was the publication plan devised at the time of registry design?

☐	**Yes**	**Comments**
☐	**No**	
☐	**N/A**	

37. Were there any issues when preparing the publication plan? If so, please specify.

☐	**Yes**	**Comments**
☐	**No**	
☐	**N/A**	

38. If you had the possibility to carry out a similar registry in the future, what would you do to improve the publication plan?

Development of the protocol and related documents

39. Were you involved in the development, revision or amendments of the protocol and/or related documents (statistical analysis plan, case report forms, etc.)? If the answer is **No**, please skip questions 40-50.

☐	**Yes**	**Comments**
☐	**No**	
☐	**N/A**	

40. Were all the members from the WC that were supposed to be involved in the development of the protocol and related documents actually involved? If not, which members were not involved and why?

☐	**Yes**	**Comments**
☐	**No**	
☐	**N/A**	

41. Were there avoidable delays during the development of the protocol and related documents? If so, please specify.

☐	**Yes**	**Comments**
☐	**No**	
☐	**N/A**	

42. Was the protocol amended? If so, please specify how many times and describe the reasons for the amendments.

☐ **Yes** **Comments**
☐ **No**
☐ **N/A**

43. Were the amendments of the protocol properly documented? If not, why not?

☐ **Yes** **Comments**
☐ **No**
☐ **N/A**

44. Were all the participants in the registry informed about the amendments in a timely manner? If not, why not?

☐ **Yes** **Comments**
☐ **No**
☐ **N/A**

45. Was the CRF developed using standard guidelines? If not, why not?

☐ **Yes** **Comments**
☐ **No**
☐ **N/A**

46. Were any patient-reported outcome or caregiver-reported outcome measures evaluated during the registry? If so, specify which one/s?

☐ **Yes** **Comments**
☐ **No**
☐ **N/A**

47. Were there any groups that did not provide patient or caregiver reported outcomes? If so, were steps to control bias taken?

☐ **Yes** **Comments**
☐ **No**
☐ **N/A**

48. Were patient/caregiver-reported outcome measures evaluated using validated instruments (validated for all languages and validated for work with children/ cognitive deficits. If they were, please specify what were the cut-offs?

☐ **Yes** **Comments**
☐ **No**
☐ **N/A**

49. Considering the international nature of the registry, were there any issues related to the translation of any of the documents? If so, please specify.

☐	**Yes**	**Comments**	
☐	**No**		
☐	**N/A**		

50. If you had the possibility to carry out a similar registry in the future, what would you do to improve the quality of the protocol and related documents?

Development of the project plan

51. Were you involved in the development of the project plan for the registry?

☐	**Yes**	**Comments**	
☐	**No**		
☐	**N/A**		

52. Do you think the project plan was clearly defined at the beginning of the project? If not, what was missing and why?

☐	**Yes**	**Comments**	
☐	**No**		
☐	**N/A**		

53. Do you think the project plan development involved all the, in your opinion, relevant members? If not, why?

☐	**Yes**	**Comments**	
☐	**No**		
☐	**N/A**		

54. In your opinion, in the project plan development was there an overrepresentation by any group, or were there members that could not really contribute?

☐	**Yes**	**Comments**	
☐	**No**		
☐	**N/A**		

55. Looking at the registry retrospectively, was the project plan sufficiently comprehensive? If not, which aspects were not adequate?

☐ **Yes**	**Comments**	
☐ **No**		
☐ **N/A**		

56. Which were, in your opinion, the most important deviations from the project plan?

57. If you had the possibility to carry out a similar registry in the future, what would you do to improve the project plan?

Development of risk management plans & risk management during the registry

58. Were you involved in the development of a risk management plan for the registry?

☐ **Yes**	**Comments**	
☐ **No**		
☐ **N/A**		

59. Did the risk management plan development involve all the, in your opinion, relevant members? If not, which members should have been involved but were not?

☐ **Yes**	**Comments**	
☐ **No**		
☐ **N/A**		

60. In your opinion, in the development or the risk management plan, was there an overrepresentation by any group, or were there members that could not really contribute?

☐ **Yes**	**Comments**	
☐ **No**		
☐ **N/A**		

61. Looking at the registry retrospectively, was the risk management plan sufficiently comprehensive? If not, which aspects were not adequate?

☐	**Yes**	**Comments**	
☐	**No**		
☐	**N/A**		

62. Looking at the registry retrospectively, were all the possible risks correctly foreseen in the risk management plan? If not, which risks were not foreseen?

☐	**Yes**	**Comments**	
☐	**No**		
☐	**N/A**		

63. Were all the risks correctly addressed during the project? If not, which risks were not addressed?

☐	**Yes**	**Comments**	
☐	**No**		
☐	**N/A**		

64. If you had the possibility to carry out a similar registry in the future, what would you do to improve the risk management plan?

Identification of issues during the operation of the registry

Issues related to patient recruitment or retention

Barriers to patient recruitment/retention

65. Do you think problems related to **patient recruitment** was an issue for the validity of the results of the core registry? Why?

☐	**Yes**	**Comments**	
☐	**No**		
☐	**N/A**		

66. Do you think problems related to **patient retention** was an issue for the validity of the results of the core registry? Why?

☐	**Yes**	**Comments**	
☐	**No**		
☐	**N/A**		

67. Please rate from 0 (not at all) to 5 (very much) how much the following issues affected, in your opinion, the recruitment or retention of patients **in the core registry** in a negative way:

**Core registry**						
	**0**	**1**	**2**	**3**	**4**	**5**
Lack of awareness of the registry						
Distrust in the sponsor, the registry leaders or the investigators						
Administrative burdens						
Perception on burdensomeness of data collection/visit scheduling						
Lack of interest in the nature of the registry/perception of low relevance or low benefits for the practice or for the patients						
Economic obstacles						
Distance between site and residence of the patients						
Ethnic/cultural views						
Language/literacy barriers						
Insufficient incentives for recruiting						
Other:						
Other:						

Comments

68. Do you think problems related to patient recruitment was an issue for the validity of the results of **the research projects**? Why?

Yes **Comments**
No
N/A

69. Do you think problems related to **patient retention** was an issue for the validity of the results of **the research projects**? Why?

Yes **Comments**
No
N/A

70. Please rate from 0 (not at all) to 5 (very much) how much the following issues affected, in your opinion, the recruitment or retention of patients in the research projects in a negative way:

**Research projects**						
	**0**	**1**	**2**	**3**	**4**	**5**
Lack of awareness of the registry						
Distrust in the sponsor, the registry leaders or the investigators						
Administrative burdens						

Perception on burdensomeness of data collection/visit scheduling						
Lack of interest in the nature of the registry/perception of low relevance or low benefits for the practice or for the patients						
Economic obstacles						
Distance between site and residence of the patients						
Ethnic/cultural views						
Language/literacy barriers						
Insufficient incentives for recruiting						
Other:						
Other:						

Comments

Evaluation of success of patient recruitment strategies

71. Which strategies were used to improve recruitment (e.g. phone contacts, targeted mailing, spreading digital or printed material through patient associations, proposal of participation in scheduled visits, etc.)?

72. Do you think the strategies used to improve recruitment were useful? If not, why not?

- ☐ **Yes**
- ☐ **No**
- ☐ **N/A**

Comments

73. Which recruitment strategies would you use again? Why?

74. If you had the possibility to carry out a similar registry in the future, what would you do to improve recruitment?

Evaluation of success of patient retention strategies

75. Which strategies were used to improve patient retention (e.g. phone or mail reminders, patient education strategies, travel and/or meal vouchers, etc.)?

76. Do you think the strategies used to improve patient retention were useful? If not, why not?

☐ **Yes** **Comments**
☐ **No**
☐ **N/A**

77. Which patient retention strategies would you use again? Why?

78. If you had the possibility to carry out a similar registry in the future, what would you do to improve patient retention?

Evaluation of center/physician or patient selection bias

79. Do you think **center/physician selection bias** (e.g. inclusion only of renowned physicians treating patients in large hospitals, exclusion of specialties of interest, over or under-sampling in particular sites or locations, etc.) was an issue in the core registry or in any of the research projects? Why?

☐ **Yes** **Comments**
☐ **No**
☐ **N/A**

80. If you had the possibility to carry out a similar registry in the future, how would you reduce center/physician selection bias?

81. Do you think **patient selection bias** (e.g. exclusion of most severe cases, exclusion of patients with affected parents, etc.) was an issue in the core registry or in any of the research projects? Why?

☐ **Yes**	**Comments**	
☐ **No**		
☐ **N/A**		

82. If you had the possibility to carry out a similar registry in the future, how would you reduce patient selection bias?

Issues related to data collection & quality assurance

Issues related to data collection

83. Do you think problems related to accuracy during data collection were an issue for the validity of the results of the core registry or of any of the research projects? Why?

☐ **Yes**	**Comments**	
☐ **No**		
☐ **N/A**		

84. Please rate from 0 (not at all) to 5 (very much) how much the following issues affected, in your opinion, the quality of data collection in a negative way:

**Issues affecting data collection**	**0**	**1**	**2**	**3**	**4**	**5**
Inadequacy of the database						
Inadequacy of case report forms (CRF)						
Lack of consistency between the data system and the CRF						
Lack of integration of the data collection procedures within the day-to-day clinical practice						
Lack of clarity of the protocol inclusion/exclusion criteria						
Lack of difference between mandatory and optional fields						
Inconsistencies in data collection/entry						
Bad translation of materials						
Lack of training						
Lack of human resources to assess quality in data collection						
Lack of technical resources to assess quality in data collection						
Other:						
Other:						

Comments:

Identification of quality issues & timing for detection

85. Please, fill the following table out:

**Type of issue detected**					
Were there any…		Yes	No	N/A	Which issue?
1	… confidentiality issues?				
2	… issues regarding informed consents?				
3	… issues regarding baseline characteristics reporting?				
4	… issues regarding adverse events detection & reporting?				
5	… issues related to data corrections and changes (e.g. lack of explanations, obscuration of original entries, etc.)?				
6	… other issues?				
**Timing for QA issues detection**					
Were there any issues detected…		Yes	No	N/A	Which issue?
1	… before opening the registry?				
2	… during the registry?				
3	… after the registry closure?				

86. Were the quality issues concentrated in a few sites/countries/regions or spread evenly across all the sites?

Yes **Comments**
No
N/A

87. Were there any systematic errors that could have been prevented by training (e.g. educating data managers to follow consistent rules during data collection and entry)? If so, please specify.

Yes **Comments**
No
N/A

88. Were there any systematic errors that could have been prevented by improving the quality of the database or the CRFs? If so, please specify.

☐ **Yes**	**Comments**	
☐ **No**		
☐ **N/A**		

89. Regarding the CRFs, please state if there were any of the following issues:

**Type of issue detected**					
Were there …		Yes	No	N/A	Specify
1	… unclear questions?				
2	… issues related to use of acronyms or abbreviations?				
3	… issues related to the lack of conditional field systems/branching logics?				
4	… issues related to the lack of a logical order for the questions?				

90. Were there inconsistencies during query solving and/or data cleaning? If so, please specify.

☐ **Yes**	**Comments**	
☐ **No**		
☐ **N/A**		

91. Which strategies were used to improve data quality (e.g. electronic CRF, in-site data manager, monitoring visits, inspections, training, keeping laboratory records, limiting expected ranges, setting double entry for key data variables, real-time data validation, periodic audits, etc.)?

92. Do you think the strategies adopted were enough to ensure data quality? If not, why not?

☐ **Yes**	**Comments**	
☐ **No**		
☐ **N/A**		

93. If you had the possibility to carry out a similar registry in the future, what would you do to improve data quality?

94. Do you think problems related to data completeness or missing data handling were an issue for the validity of the results of the core registry or of any of the research projects? Why?

☐ **Yes**	**Comments**	
☐ **No**		
☐ **N/A**		

95. In the perspective of data quality, was there a clear distinction between "essential" and "non-essential" variables? If not, why not?

☐ **Yes**	**Comments**	
☐ **No**		
☐ **N/A**		

96. If data collection was based on electronic data capture, were there automatic rules to prevent missing inputs for the "essential" variables? If not, why not?

☐ **Yes**	**Comments**	
☐ **No**		
☐ **N/A**		

97. Which were the variables with most missing data? Why?

98. Which were the variables with less missing data? Why?

99. Were there any clear patterns for missing data (e.g. based on baseline characteristics, center, type of variable, type of manifestation, etc)? If so, please specify.

☐ **Yes**	**Comments**	
☐ **No**		
☐ **N/A**		

Issues related to budget

100. Were you involved in budget planning, allocation and/or control? If the answer is **No**, please skip questions 101-112.

☐ **Yes**	**Comments**	
☐ **No**		
☐ **N/A**		

101. Were there any difficulties when finding budget for the design and/or development of the registry? If so, please specify.

- [ ] **Yes** **Comments**
- [ ] **No**
- [ ] **N/A**

102. Were there any issues when deciding about the costs to be paid by Novartis or by the participating centers? If so, please specify.

- [ ] **Yes** **Comments**
- [ ] **No**
- [ ] **N/A**

103. Within Novartis, who approved the split of costs to be paid by Novartis or by the participating centers?

- [ ] **Yes** **Comments**
- [ ] **No**
- [ ] **N/A**

104. Was the design of the trial limited by budget constraints? If so, please specify.

- [ ] **Yes** **Comments**
- [ ] **No**
- [ ] **N/A**

105. Were the actual costs coherent with the planned budget? If not, why?

- [ ] **Yes** **Comments**
- [ ] **No**
- [ ] **N/A**

106. If you had the possibility to carry out a similar registry in the future, what would you do to improve budget planning (i.e. reducing the difference between planned vs real)?

107. Which were the main deviations between the planned budget vs the real costs?

108. Which were the major cost drivers of total expenditure?

109. Do you think the major cost drivers were critical for the registry development? Why?

Yes **Comments**
No
N/A

110. When breaking down the budget items, was there any item that added no value or added low value to the overall quality of the registry? If so, please specify.

Yes **Comments**
No
N/A

111. Were there any missed opportunities to reduce costs? If so, please specify.

Yes **Comments**
No
N/A

112. If you had the possibility to carry out a similar registry in the future, what would you do to increase the quality/cost ratio of the registry?

Related to project management

113. Were you involved in any project management tasks?

Yes **Comments**
No
N/A

Ownership & accountability

114. Would you consider that all the members of the team took their share of ownership within the project? If not, why not?

☐ **Yes** **Comments**
☐ **No**
☐ **N/A**

115. Which were the measures directed to help the members of the team to take ownership over their work? Did these measures work? If not, why not?

116. What would you do to improve ownership and accountability within the team in similar projects?

Coordination

117. Were there any problems related to poor coordination within the core registry or within the research projects? If so, please specify.

☐ **Yes** **Comments**
☐ **No**
☐ **N/A**

118. Were there any measures to improve coordination? If so, please specify.

☐ **Yes** **Comments**
☐ **No**
☐ **N/A**

119. Did they work? If not, why not?

☐ **Yes** **Comments**
☐ **No**
☐ **N/A**

120. What would you do to improve coordination within the team in similar projects?

Estimation of the use of resources/duration/complexity

121. Was the estimation of needed resources adequate? If not, why not?

☐ **Yes**	**Comments**	
☐ **No**		
☐ **N/A**		

122. Which were the most important issues that caused deviation from timelines? Please describe how severe they were.

123. Do you think any of the deviations from timelines could have been avoided? How?

☐ **Yes**	**Comments**	
☐ **No**		
☐ **N/A**		

124. Was the complexity of the project underestimated? Why?

☐ **Yes**	**Comments**	
☐ **No**		
☐ **N/A**		

125. If you had the possibility to carry out a similar registry in the future, what would you do to reduce the deviation between expected and real estimations regarding use of resources, duration & complexity?

Issues during data analysis

126. Were you involved in the development of the **statistical analysis plan**?

☐ **Yes**	**Comments**	
☐ **No**		
☐ **N/A**		

127. Were you involved in data analysis or in the development of any of the **registry reports**?

☐	**Yes**	**Comments**	
☐	**No**		
☐	**N/A**		

Identification of sources of bias

128. Do you think the risk of bias was properly assessed prior to the registry? If not, why?

☐	**Yes**	**Comments**	
☐	**No**		
☐	**N/A**		

129. Please rate the risk (inexistent, low, medium or high) of the following types of biases in the TOSCA registry:

	**Inexistent risk**	**Low risk**	**Medium risk**	**High risk**
Selection bias:<br>• unclear inclusion/exclusion criteria<br>• registry population is not a random selection from the target population<br>• …				
Information bias:<br>• selective recall<br>• inconsistent data collection<br>• wrong or inexact data recording<br>• …				
Measurement errors<br>• faulty or inaccurate measurements<br>• misclassification of outcomes<br>• …				
Other (please specify):				

130. Do you think any of the biases listed above was an issue compromising the validity of the results? If yes, why?

☐	**Yes**	**Comments**	
☐	**No**		
☐	**N/A**		

131. If you had the possibility to carry out a similar registry in the future, what would you do to reduce bias?

Treatment of missing data

132. Do you think treatment of missing data or the lack thereof was an issue in this registry? Why?

- [ ] **Yes** **Comments**
- [ ] **No**
- [ ] **N/A**

133. Was the amount of missing data reported for each variable? If not, why not?

- [ ] **Yes** **Comments**
- [ ] **No**
- [ ] **N/A**

134. Were the differences between individuals with complete data and those with incomplete data assessed? If not, why not?

- [ ] **Yes** **Comments**
- [ ] **No**
- [ ] **N/A**

135. Were all missing data given an explanation? If not, why not?

- [ ] **Yes** **Comments**
- [ ] **No**
- [ ] **N/A**

136. Were the statistical methods for handling missing data clearly stated in the statistical analysis plan?

- [ ] **Yes** **Comments**
- [ ] **No**
- [ ] **N/A**

137. Were missing data consistently handled throughout the registry? If not, why not?

- [ ] **Yes** **Comments**
- [ ] **No**
- [ ] **N/A**

138. Were there any specific considerations for missing data handling due to rarity of the disease with a high variability? If not, why not?

☐ **Yes** ☐ **No** ☐ **N/A**	**Comments**	

139. If you had the possibility to carry out a similar registry in the future, what would you improve missing data handling?

Appropriateness of time horizon & planned interim analysis

140. Was the time horizon of the registry adequate? If not, why?

☐ **Yes** ☐ **No** ☐ **N/A**	**Comments**	

141. How was the number of planned interim analysis decided? Describe if there were any specific considerations.

142. Was the number of planned interim analysis adequate? If not, why?

☐ **Yes** ☐ **No** ☐ **N/A**	**Comments**	

143. Was the timing for interim analyses adequate? If not, why?

☐ **Yes** ☐ **No** ☐ **N/A**	**Comments**	

Appropriateness of pre-specified analyses

144. If you had the possibility to carry out a similar registry in the future, would you redefine any of the main pre-planned subgroup analyses (*i.e.* age, country, mutation profile)?

☐ **Yes**	**Comments**	
☐ **No**		
☐ **N/A**		

145. Regarding age-groups, a large proportion of patients who turned 18 during the registry filled questionnaires for pediatric patients throughout the whole registry (instead of using questionnaires for adults). Do you think this was an issue? If so, how would you solve this issue in future studies?

☐ **Yes**	**Comments**	
☐ **No**		
☐ **N/A**		

146. If you had the possibility to carry out a similar registry in the future, would you perform analyses in pre-defined groups of countries? If so, which ones?

☐ **Yes**	**Comments**	
☐ **No**		
☐ **N/A**		

147. If you had the possibility to carry out a similar registry in the future, would you perform analyses in patients sharing specific groups of manifestations? If so, which ones?

☐ **Yes**	**Comments**	
☐ **No**		
☐ **N/A**		

148. If you had the possibility to carry out a similar registry in the future, would you analyze the use of treatments depending on the severity of the manifestations?

☐ **Yes**	**Comments**	
☐ **No**		
☐ **N/A**		

149. If you had the possibility to carry out a similar registry in the future, do you think it would be interesting to trace individual treatment patterns (at a patient-level)?

☐ **Yes**	**Comments**	
☐ **No**		
☐ **N/A**		

150. If you had the possibility to carry out a similar registry in the future, would you include any other analyses than those specified above?

☐ **Yes**	**Comments**	
☐ **No**		
☐ **N/A**		

Interpretation of the results

151. Do you think the lack of internal or external validity was an issue in this registry? Why?

- [ ] **Yes** **Comments**
- [ ] **No**
- [ ] **N/A**

152. If you had the possibility to carry out a similar registry in the future, what would you do to increase the internal and/or the external validity of the results?

- [ ] **Yes** **Comments**
- [ ] **No**
- [ ] **N/A**

Identification of issues related to data access

153. Were there any issues related to data access **during** the registry? Which issues?

- [ ] **Yes** **Comments**
- [ ] **No**
- [ ] **N/A**

154. Were there any issues related to data access **after** the registry? Which issues?

- [ ] **Yes** **Comments**
- [ ] **No**
- [ ] **N/A**

155. Are anonymized patient-level data available for external researchers under request? If not, why not?; If yes, under which conditions?

- [ ] **Yes** **Comments**
- [ ] **No**
- [ ] **N/A**

156. Are aggregated data available to external researchers under request? If not, why not?; If yes, under which conditions?

- [ ] **Yes** **Comments**
- [ ] **No**
- [ ] **N/A**

157. Are data in a format amenable to linkage to national/international databases?

- [ ] **Yes** **Comments**
- [ ] **No**
- [ ] **N/A**

158. According to the FAIR principles, the data stored in databases should be **F**indable, **A**ccessible, **I**nteroperable and **R**eusable to allow data exchange. In your opinion, does the TOSCA registry database fulfill these conditions? If not, why not?

☐	**Yes**	**Comments**	
☐	**No**		
☐	**N/A**		

Identification of strengths & limitations of the registry

159. Please, list the 5 strengths of the registry that are, in your opinion, the most remarkable.

- …
- …
- …
- …
- …

160. Please, list the 5 limitations of the registry that are, in your opinion, the most remarkable.

- …
- …
- …
- …
- …

Issues during the publication of the results

161. Were there any authorship conflicts? If so, how do you think they could have been avoided?

☐	**Yes**	**Comments**	
☐	**No**		
☐	**N/A**		

162. In your opinion, did all the authors of the manuscripts make substantial contributions to conception and design, and/or acquisition of data, and/or analysis and interpretation of data? If not, why?

☐	**Yes**	**Comments**	
☐	**No**		
☐	**N/A**		

163. Did all the authors of the manuscripts participated in drafting the article or revising it critically for important intellectual content? If not, why?

☐	**Yes**	**Comments**	
☐	**No**		
☐	**N/A**		

164. Did all the authors of the manuscripts give final approval of the versions that were submitted for publication? If not, why?

☐	**Yes**	**Comments**	
☐	**No**		
☐	**N/A**		

165. Were all the contributors (medical writers, statisticians, other professionals providing technical support, etc.) who did **not** meet the criteria for authorship listed in the acknowledgments?

☐	**Yes**	**Comments**	
☐	**No**		
☐	**N/A**		

166. Were there any unexpected delays in the publication plan? Why?

☐	**Yes**	**Comments**	
☐	**No**		
☐	**N/A**		

167. Were there any differences between the foreseen and the final target journals/conferences? Why?

☐	**Yes**	**Comments**	
☐	**No**		
☐	**N/A**		

168. If you had the possibility to carry out a similar registry in the future, what would you do **to improve the quality of the publications?**

169. If you had the possibility to carry out a similar registry in the future, what would you **do to increase the speediness of the publication process without affecting its quality?**

Other issues

170. Were there any issues related to differences in the regulation on the registration or use of health information in different countries? If so, please specify.

☐ **Yes** **Comments**
☐ **No**
☐ **N/A**

171. Were there any issues related to differences in transparency, property rights & data ownership requirements in different countries? If so, please specify.

☐ **Yes** **Comments**
☐ **No**
☐ **N/A**

172. Were there any issues related to the registry oversight (e.g. scientific independence, guidance, role of the stakeholders, conflict of interest, etc.)? If so, please specify.

☐ **Yes** **Comments**
☐ **No**
☐ **N/A**

173. Were there any issues regarding the translation of the ethical concerns into different legal regulation/regulatory frameworks in different countries? If yes, please specify. If not, were there any lessons learnt for future projects?

☐ **Yes** **Comments**
☐ **No**
☐ **N/A**

174. Were there any issues derived from language and cultural barriers? If so, please specify.

☐ **Yes** **Comments**
☐ **No**
☐ **N/A**

175. Were there any issues derived from differences in administrative requirements between different countries? If so, please specify.

☐ **Yes** **Comments**
☐ **No**
☐ **N/A**

176. Were there any issues derived from site dispersion? If so, please specify.

☐	**Yes**	**Comments**	
☐	**No**		
☐	**N/A**		

177. Were there any issues arising from adverse event collection and/or reporting? If so, please specify.

☐	**Yes**	**Comments**	
☐	**No**		
☐	**N/A**		

Assessment of learnings

General learning topics

178. Please, fill the following table out:

**Do you think this registry has contributed…**	**Yes**	**No, but it was intended**	**No, but it was not intended**
… to improve the knowledge on the natural history of TSC and its manifestations?			
… to improve the knowledge on the clinical management of the disease in different countries?			
… to improve epidemiological knowledge of TSC?			
… to provide data on the effectiveness & efficiency of interventions in the real world?			
… to provide data on quality of life?			
… to provide data on the safety of the interventions in patients with TSC in the real world?			
… to assess the agreement between clinical practice and guidelines?			
… to improve clinical practice?			
… to develop new clinical practice guidelines?			
… to increase disease awareness?			
… to quantify the use of resources and the burden of the disease?			
… to improve health care planning & resource allocation?			
… to trigger research questions/developing hypothesis for new research in TSC?			
… to identify patients with TSC that might benefit from certain interventions or might be included in future clinical trials?			
… to identify centers/physicians treating patients with TSC?			
… to identify useful information for the development of clinical research in TSC?			

… to identify useful information for the development of clinical research in other rare diseases?			
… to identify useful information for the development of studies involving large/diverse geographic areas?			
… to identify useful information for the development of studies in pediatric patients?			
… to foster the communication between TSC experts?			
… to foster the communication between TSC experts and Novartis?			
… to foster the communication between TSC experts and patients?			
… to foster the communication between TSC patients and Novartis?			
… to facilitate market access for Votubia?			
Other (please, specify):			
Other (please, specify):			
Other (please, specify):			

Comments

Value of the registry organization

Inclusion of patients in the SAB and in the WC

179. Please, rate from 0 (not at all) to 5 (very much) the relevance of the participation of **patient representatives** in the SAB and in the WC:

	**0**	**1**	**2**	**3**	**4**	**5**
To include variables or analyses of interest for the patients						
To improve recruitment						
To improve maintenance of patients in the registry						
To improve the interpretation of the results						
To improve the quality of the publications						
To increase trust in the registry						
To provide feedback on patient issues or concerns about the registry						
To facilitate communication about the registry’s purpose and value to patient advocacy groups						
To increase public awareness on the disease						
Other (please, specify):						

180. Do you think including patient representatives in the SAB and in the WC was positive to improve the overall quality of the registry design? Why?

☐	**Yes**	**Comments**	
☐	**No**		
☐	**N/A**		

181. Do you think including patient representatives in the SAB and in the WC was positive to improve the overall quality of the registry results? Why?

☐	**Yes**	**Comments**	
☐	**No**		
☐	**N/A**		

182. Were there any ethical issues related to the inclusion of patient representatives in the SAB and in the WC? If yes, which ones?

☐	**Yes**	**Comments**	
☐	**No**		
☐	**N/A**		

183. Were there confidentiality issues related to the inclusion of patient representatives in the SAB and in the WC? If yes, which ones?

☐	**Yes**	**Comments**	
☐	**No**		
☐	**N/A**		

184. If you had the possibility to carry out a similar registry, would you increase or decrease the number of patient representatives in the SAB or in the WC? Why?

☐	**Yes**	**Comments**	
☐	**No**		
☐	**N/A**		

Inclusion of clinicians in the SAB and in the WC

185. Please, rate from 0 (not at all) to 5 (very much) the relevance of the participation of **TSC experts** in the SAB and in the WC:

	**0**	**1**	**2**	**3**	**4**	**5**
To propose the collection of variables and analyses of medical interest						
To improve the design of the protocol						
To improve the design of the CRFs						
To keep alignment with the definitions of the current clinical manifestations/variables						
To improve recruitment						

To improve maintenance of patients in the registry						
To increase awareness among the medical community						
To increase public awareness on the disease						
To provide interpretations for the results						
To improve the quality of abstracts or manuscripts						
To guarantee registry transparency						
To guarantee registry independence						
Other (please, specify):						

186. Do you think including TSC experts in the SAB and in the WC was positive to improve the overall quality of the registry design? Why?

	**Yes**	**Comments**	
	**No**		
	**N/A**		

187. Do you think including TSC experts in the SAB and in the WC was positive to improve the overall quality of the registry results? Why?

	**Yes**	**Comments**	
	**No**		
	**N/A**		

188. If you had the possibility to carry out a similar registry, would you increase or decrease the number of TSC experts in the SAB and in the WC? Why?

	**Yes**	**Comments**	
	**No**		
	**N/A**		

Inclusion of members from the pharmaceutical industry in the SAB and in the WC

189. Please, rate from 0 (not at all) to 5 (very much) the relevance of the participation of **members from the pharmaceutical industry** in the SAB and in the WC:

	**0**	**1**	**2**	**3**	**4**	**5**
To propose the collection of variables and analyses of medical interest						
To provide support in the design of the protocol and/or CRFs						
To provide technical support for data collection						
To serve as a liaison between the members of the committees and between the committees						
To ensure compliance with agreed timelines						
To ensure quality standards for collecting, cleaning, storing, monitoring, reviewing, and reporting data						
To ensure the compliance of ethical/legal standards						

To alleviate administrative burdens						
To provide technical or financial support for the dissemination of the results in scientific venues						
To provide technical or financial support for the publication of the results						
Other (please, specify):						

190. Were different pharmaceutical companies involved in the registry? If not, please describe if in your opinion, this would have been positive or negative for the registry.

☐ **Yes**	**Comments**	
☐ **No**		
☐ **N/A**		

191. Do you think including members from the pharmaceutical industry in the SAB and in the WC was positive to improve the overall quality of the registry design? Why?

☐ **Yes**	**Comments**	
☐ **No**		
☐ **N/A**		

192. Do you think including members from the pharmaceutical industry in the SAB and in the WC was positive to improve the overall quality of the registry results? Why?

☐ **Yes**	**Comments**	
☐ **No**		
☐ **N/A**		

193. If you had the possibility to carry out a similar registry, would you increase or decrease the number of members from the pharmaceutical industry in the SAB and in the WC? Why?

☐ **Yes**	**Comments**	
☐ **No**		
☐ **N/A**		

Pitfalls and learning opportunities emerged from the integration of research projects within the TOSCA registry

194. Overall, do you think it was appropriate to include research projects within the structure of the TOSCA registry? Why?

☐ **Yes**	**Comments**	
☐ **No**		
☐ **N/A**		

195. Please, list what you consider the most relevant pitfalls and opportunities derived from including the research projects within the TOSCA registry:

Pitfalls derived from including research projects within the TOSCA registry
• …<br>• …<br>• …<br>• …
Opportunities derived from including research projects within the TOSCA registry
• …<br>• …<br>• …<br>• …

196. Were the research projects approved separately (i.e. in different moments) from the core registry? In your opinion, was that convenient? Why?

☐	**Yes**	**Comments**	
☐	**No**		
☐	**N/A**		

197. Were the documents (protocol, CRF, etc.) for the research projects elaborated separately from those of the core project? In your opinion, was that convenient? Why?

☐	**Yes**	**Comments**	
☐	**No**		
☐	**N/A**		

198. Was data collection for the research projects performed separately from the core registry? In your opinion, was that convenient? Why?

☐	**Yes**	**Comments**	
☐	**No**		
☐	**N/A**		

199. In your opinion, were time and resources adequately managed in the research projects? Why?

☐	**Yes**	**Comments**	
☐	**No**		
☐	**N/A**		

200. In your opinion, were there variables included in the research projects that should have been included in the core registry? Which ones?

☐	**Yes**	**Comments**	
☐	**No**		
☐	**N/A**		

201. In your opinion, were there variables included in the core registry that should have been included only in the research projects? Which ones?

☐	**Yes**	**Comments**	
☐	**No**		
☐	**N/A**		

202. Did the quality/completeness of the data from the research projects differ from that of the core project?

☐	**Yes**	**Comments**	
☐	**No**		
☐	**N/A**		

203. Do you think the number of patients included in the research projects was sufficient to answer questions of clinical relevance?

☐	**Yes**	**Comments**	
☐	**No**		
☐	**N/A**		

204. In your opinion, were the results/ conclusions observed in the research projects translatable to the population included in the TOSCA?

☐	**Yes**	**Comments**	
☐	**No**		
☐	**N/A**		

205. In your opinion, were the results/ conclusions observed in the research projects translatable to patients in the real world?

☐	**Yes**	**Comments**	
☐	**No**		
☐	**N/A**		

206. In your opinion, were there any results of the research projects particularly striking or relevant? Which ones?

☐	**Yes**	**Comments**	
☐	**No**		
☐	**N/A**		

207. Did any hypotheses for new projects emerge from the research projects? Which ones?e

☐	**Yes**	**Comments**	
☐	**No**		
☐	**N/A**		

208. In your opinion, were the results of the research projects adequately disseminated? Why?

☐	**Yes**	**Comments**	
☐	**No**		
☐	**N/A**		

Pitfalls and learning opportunities emerged from the integration of a Votubia PASS within the TOSCA registry

209. Overall, do you think it was appropriate to integrate the Votubia PASS within the TOSCA registry? Why?

☐	**Yes**	**Comments**	
☐	**No**		
☐	**N/A**		

210. Please, list the most relevant pitfalls and opportunities derived from integrating the Votubia PASS within the TOSCA registry :

Pitfalls derived from integrating the Votubia PASS within the TOSCA registry
• …<br>• …<br>• …<br>• …
Opportunities derived from integrating the Votubia PASS within the TOSCA registry
• …<br>• …<br>• …<br>• …

211. Was the PASS approved separately (i.e.in different moments) from the core registry? In your opinion, was that convenient?

☐	**Yes**	**Comments**	
☐	**No**		
☐	**N/A**		

212. Were the documents (protocol, CRF, etc.) for the PASS elaborated separately from those of the core project? In your opinion, was that convenient?

☐	**Yes**	**Comments**	
☐	**No**		
☐	**N/A**		

213. Was data collection for the PASS done separately from the core registry? In your opinion, was that convenient?

☐	**Yes**	**Comments**	
☐	**No**		
☐	**N/A**		

214. In your opinion, were time and resources adequately managed in the PASS ? Why?

☐	**Yes**	**Comments**	
☐	**No**		
☐	**N/A**		

215. In your opinion, were there variables included in the PASS that should have been included in the core registry? Which ones?

☐	**Yes**	**Comments**	
☐	**No**		
☐	**N/A**		

216. In your opinion, were there variables included in the core registry that should have been included only in the PASS ? Which ones?

☐	**Yes**	**Comments**	
☐	**No**		
☐	**N/A**		

217. Did the quality/completeness of the data from the PASS differ from that of the core registry? Why?

☐	**Yes**	**Comments**	
☐	**No**		
☐	**N/A**		

218. Do you think the number of patients included in the PASS was adequate? Why?

☐	**Yes**	**Comments**	
☐	**No**		
☐	**N/A**		

219. In your opinion, were the results observed in the PASS translatable to the population included in the TOSCA? If not, why?

☐	**Yes**	**Comments**	
☐	**No**		
☐	**N/A**		

220. In your opinion, were the results/conclusions observed in the PASS translatable to patients in the real world? If not, why?

☐	**Yes**	**Comments**	
☐	**No**		
☐	**N/A**		

221. In your opinion, were there any results of the PASS particularly striking or relevant? Which ones?

☐	**Yes**	**Comments**	
☐	**No**		
☐	**N/A**		

222. Did any hypothesis for new projects emerge from the PASS? Which ones?

☐	**Yes**	**Comments**	
☐	**No**		
☐	**N/A**		

223. In your opinion, were the results of the PASS adequately disseminated? Why?

☐	**Yes**	**Comments**	
☐	**No**		
☐	**N/A**		

224. In your opinion, did the interaction for the health authorities during the PASS benefit the development of the TOSCA registry? If yes, describe the lessons learned from these interactions.

☐	**Yes**	**Comments**	
☐	**No**		
☐	**N/A**		

Additional comments

Please use this box to comment on any topic related to issues or learnings from the TOSCA registry and to provide further clarifications on your views on the registry.

THANK YOU VERY MUCH FOR FILLING THIS QUESTIONNAIRE OUT.

Your views help in learning for future research
